# AHR Regulates NK Cell Migration *via* ASB2–Mediated Ubiquitination of Filamin A

**DOI:** 10.3389/fimmu.2021.624284

**Published:** 2021-02-24

**Authors:** June Ho Shin, Uriel Y. Moreno-Nieves, Luhua H. Zhang, Chen Chen, Amera L. Dixon, Miles H. Linde, Emily M. Mace, John B. Sunwoo

**Affiliations:** ^1^ Department of Otolaryngology - Head and Neck Surgery, Stanford Cancer Institute and Institute for Stem Cell Biology and Regenerative Medicine, Stanford University School of Medicine, Stanford, CA, United States; ^2^ Department of Pediatrics, Columbia University Irving Medical Center, New York, NY, United States

**Keywords:** tumor, migration, filamin A, ASB2, AHR, NK cells

## Abstract

Natural killer (NK) cells are effector cells of the innate immune system involved in defense against virus-infected and transformed cells. The effector function of NK cells is linked to their ability to migrate to sites of inflammation or damage. Therefore, understanding the factors regulating NK cell migration is of substantial interest. Here, we show that in the absence of aryl hydrocarbon receptor (AHR), a ligand-activated transcription factor, NK cells have reduced capacity to migrate and infiltrate tumors *in vivo*. Analysis of differentially expressed genes revealed that ankyrin repeat and SOCS Box containing 2 (*Asb2*) expression was dramatically decreased in *Ahr* ^–/–^ NK cells and that AhR ligands modulated its expression. Further, AhR directly regulated the promoter region of the *Asb2* gene. Similar to what was observed with murine *Ahr* ^–/–^ NK cells, *ASB2* knockdown inhibited the migration of human NK cells. Activation of AHR by its agonist FICZ induced ASB2-dependent filamin A degradation in NK cells; conversely, knockdown of endogenous *ASB2* inhibited filamin A degradation. Reduction of filamin A increased the migration of primary NK cells and restored the invasion capacity of AHR-deficient NK cells. Our study introduces AHR as a new regulator of NK cell migration, through an AHR-ASB2-filamin A axis and provides insight into a potential therapeutic target for NK cell-based immunotherapies.

## Introduction

Natural killer (NK) cells are innate lymphocytes that belong to the Group 1 innate lymphoid cell (ILC) family and are able to respond rapidly to virally infected or transformed cells (1). The function of NK cells is controlled by an array of germline-encoded receptors that enable them to sample the microenvironment and rapidly exert their effector functions without the need of prior stimulation (2).

NK cells are found in the peripheral blood and within tissues, where they can be classified as circulating or tissue-resident cells depending on their phenotype and function (3). During the inflammatory response, NK cells are rapidly mobilized to the site of inflammation and constitute one of the earliest effector cells in place (4).

Trafficking of NK cells from blood into tissue compartments, including the tumor microenvironment, is regulated by chemokines and cytokines. NK cells express several chemokine receptors, such as CCR5, CCR7, CXCR3, CXCR4, CXCR6, CCR7 [reviewed in (5)], whose expression is essential for the tissue tropism of NK cells and their interaction with other cell types. NK cells respond to several cytokines and some of them modulate their migratory capacity. For example, IL-2 and IL-15 induce homing of NK cells to tissues (6), whereas TGF-β impairs their migration (7, 8). Transcription factors, like T-bet (9), also regulate the ability of NK cells to migrate.

Aryl hydrocarbon receptor (AHR) is a ubiquitously expressed ligand-activated transcription factor that responds to endogenous and exogenous ligands, such as 6-formylindolo[3,2- b]carbazole (FICZ) and TCDD (10, 11), respectively. AHR binds to dioxin response element (DRE) sequences in the regulatory regions of target genes and modulates their expression (12).

AHR is expressed and exerts biological functions in several cell types, including immune cells (13–15). In immune cells, AHR is involved in a variety of processes, such as the xenobiotic response, inflammatory response, antioxidant response, estrogen response, differentiation and the cell cycle [reviewed in (13–15)]. Concerning NK cells, AHR is involved in the regulation of the plasticity between ILC3 and CD56^bright^ NK cells (16), the homeostasis of liver-resident NK cells (17), the anti-tumor response (18), cytokine production (19, 20), as well as receptor repertoire expression, including the expression of trafficking receptors (19).

Regarding the migration of immune cells, it was reported that AHR regulates the migration of dendritic cells (21) and regulatory T cells (22). However, the effect of AHR modulation in other immune cell types, including NK cells, has not been fully assessed. In a previous study, we observed that AHR-deficiency was associated with low infiltration of lymphocytes into the tumor microenvironment (18). Here, we investigated whether AHR regulates the migration of NK cells. We found that AHR regulates the expression of ankyrin repeat and SOCS Box containing 2 (ASB2), which encodes the specificity subunit of a multimeric E3 ubiquitin ligase (23), and that ASB2 regulates the ubiquitination and proteasome degradation of filamin A, which in turn modulates NK cell migration.

## Materials and Methods

### Mice

C57BL/6, *Ahr* ^+/−^, and B6.*Rag1* ^−/−^ mice were obtained from Jackson Laboratory. B10;B6.Rag2^−/−^γc^−/−^ mice were obtained from Taconic. *Ahr^−/−^* mice were established by breeding *Ahr^+/−^* mice and confirmed by the genotyping strategy outlined by the vendor. NSG mice in a C57BL/6 background were a gift from Dr. Irving L. Weissman (Stanford). Mice were kept under specific pathogen-free conditions, and 6–8 week-old mice were used for the experiments. All animal procedures were performed in accordance with protocols approved by the Administrative Panel on Laboratory Animal Care at Stanford University (Stanford, CA).

### Cells and Culture

To obtain mouse splenic NK cells, spleens were harvested, mechanically dissociated and filtered through a 70 µm cell strainer (Falcon; Cat# 352350) to obtain a single-cell suspension. Mouse NK cells were isolated by negative isolation from the spleen single-cell suspension (STEMCELL Technologies; Cat# 19855), according to manufacturer’s protocol, and cultured in 1,000 U/mL of IL-2 (NCI BRB Preclinical Repository). To obtain human primary NK cells, blood from healthy donors was obtained from the Stanford Blood Center, in accordance with a protocol approved by the IRB at Stanford University, and NK cells were enriched using RosetteSep™ NK Cell Enrichment Cocktail (STEMCELL Technologies; Cat# 15065) according to manufacturer’s instructions. Primary NK cells were cultured in RPMI 1640 (Corning; Cat# 10-040-CV) supplemented with 10% heat-inactivated Fetal Bovine Serum (Omega Scientific; Cat# FB-21), 1% Pen-Strep (Gibco; Cat# 15140-122), 55 µM 2-Mercaptoethanol (Gibco; Cat# 21985-023), 1x MEM Non-Essential Amino-Acids (Gibco; Cat# 11140-050), 1 mM Sodium Pyruvate (Gibco; Cat# 11360-070), and 10 mM HEPES (Gibco; Cat# 25-060-Cl).

The human HNSCC cell line UM-SCC-103 was kind gifts from Dr. Suzanne Gollin Theresa Whiteside (University of Pittsburgh, PA) and SCC-4 cell line was obtained from ATCC. Cells were maintained in complete DMEM/F12 medium (DMEM:F12 with Glutamax [Gibco, Invitrogen, CA] containing: 10% heat-inactivated FBS [Omega Scientific, CA], 100 IU/ml penicillin and 100 μg/ml streptomycin [Gibco, Invitrogen, CA]). The MOC2 murine oral SCC cell lines were developed by Dr. Ravindra Uppaluri at Washington University. The HEK-293 cell line was obtained from ATCC and maintained in complete DMEM medium. The NK-92MI human NK cell line was cultured in RPMI 1640 with L-glutamine supplemented with 20% heat-inactivated FBS, 1% Pen-Strep, 0.2 mM i-inositol (Sigma-Aldrich; Cat# I5125), 20 mM folic acid (Sigma-Aldrich; Cat# F8758), 0.1 mM 2-Mercaptoethano and 1x MEM Non-Essential Amino-Acids. Culture media were renewed every 2–3 days depending on cell density, and subculture was conducted when confluence was reached.

For some experiments, to assess the involvement of AhR, media was supplemented with either DMSO (ATCC; Cat# 4-X), 200 nM FICZ (ENZO; Cat# BML-GR206-0100) or 3 µM CH-223191 (Sigma-Aldrich; Cat# C8124-5MG). For sorting, NK cells were resuspended on culture media and CD56^bright^ and CD56^dim^ NK cells were sorted on a FACS Aria II instrument (BD), as previously described (19). Purities of sorts were >95%.

### Luciferase Assay

*mAsb2* promoter region (-973/-1 from ATG) was PCR cloned (F; 5’-TCAGATAGGCTGGTGAATGATGC-3’, R; 5’-CTCGGCCACCTCTCCTCCAGCTT-3’) with *mAsb2* BAC clone (RP23-213c17, bacpac, CA) and subcloned into EcoRI/HindIII of pBV-Luc reporter vector (Addgene, MA). *mAhr* expression vector was PCR cloned with cDNA clone into EcoRI site of pIRES2-EGFP expression vector (Clontech, CA). hAhR expression vector (pCMV6-hAhR) was purchased from OriGene (MD, USA). To test promoter activity, pBV-Luc reporter vector containing *Asb2* promoter and *mAhr* or *hAHR* expression vectors were co-transfected into HEK-293 cells with Lipofectamin2000 (Invitrogen, CA). After 48 h, cells were collected and the luciferase activity was measured. Renilla luciferase was used for normalization.

To examine the activity of AHR ligands and cytokines, Cignal XRE Reporter stable transfected NK-92MI cells (PNAS, 2013) were used. For reporter gene analysis, NK-92MI reporter cells (1×10^5^) were plated onto 24-well plates and stimulated with AHR ligands. Cells were harvested at specific time points after treatment and firefly luciferase activity was measured by using a Dual-Luciferase Assay System (Promega; Cat# E1910). The fluorescence intensity was measured by using FLUOstar OPTIMA (BMG Labtech).

### ChIP Assay

Murine splenic NK cells were stimulated by IL-2 (1,000 U/mL, NCI BRB Preclinical Repository) for 10 days, fixed, lysed. and used for chromatin-immunoprecipitation assay (Abcam; Cat# ab500) according to manufacturer’s protocol. DNA fragments were immunoprecipitated using anti-AHR antibody (Abcam; Cat# ab2769), anti-H3 (positive control) antibody, and protein A bead only as a negative control. The immunoprecipitated DNA was amplified by PCR for 30 cycles using specific promoter primers (F; 5’-GCTACTCATGCAGAAGACCCA-3’ and R; 5’-TCCCTGTGAGGAAACCGAAC-3’) for *Asb2* gene.

### Tumor Infiltration Assay

Mouse MOC2 oral squamous cells (1x10^6^) were injected into the sub-cutaneous compartment of NSG mice. When tumor diameter reached 5 mm, purified splenic NK cells from *Ahr^+/+^* or *Ahr^–/–^* mice were injected *via* tail vain (5x10^6^ cells/mice). Prior injection, NK cells were cultured in the presence of IL-2 and labeled with Vybrant^®^ DiI Cell-Labeling Solution (Invitrogen; Cat# V22885), according to the manufacturer’s instructions.

Similarly, human SCC-4 or UM-SCC-103 oral squamous cells (1x10^6^) were injected into the sub-cutaneous compartment of NSG mice. When tumor diameter reached 5 mm, NK-92MI expressing GFP (NK-92MI-GFP), stably transfected with *ASB2* shRNA, *AHR* shRNA or *AHR/FLNA* double shRNA, were injected *via* tail vain (5x10^6^ cells/mice).

After 24 h, tumors were harvested and dissociated, and NK cell infiltration was quantified by flow cytometry analysis.

### Tumor Dissociation

Tumors were minced and digested in 300 U/ml collagenase and 100 U/ml hyaluronidase (STEMCELL Technologies; Cat# 07912) in culture media (DMEM/F12 medium with 10% FBS, 2 mmol/L L-glutamine, and 1% penicillin-streptomycin-amphotericin B [MP Biomedicals; Cat# ICN1674049]). The tumor digestion was pipetted every 15 min and incubated at 37°C for 3 h, until a single-cell suspension was obtained. The dissociated cells were spun down and resuspended in Trypsin-EDTA (STEMCELL Technologies; Cat# 07901) for 5 min, then further dissociated with 5 U/ml dispase (STEMCELL Technologies; Cat# 07923) and 0.1 mg/ml DNase I (STEMCELL Technologies; Cat# 07900) for 1 min. Then cells were filtered through a 40 µm cell strainer and erythrocytes were lysed with ACK lysing buffer (Lonza; Cat# 10-548E).

### qRT-PCR and Microarrays

RNA was extracted with the RNeasy mini kit (QIAGEN) and cDNA made with the Maxima First Strand cDNA Kit (Thermo Scientific). Quantitative gene expression was performed using the Taqman Gene Expression Assay with the recommended primers (Life Technologies). Each gene expression assessment was measured in triplicates. Gene expression was normalized to control for *HPRT1* expression then shown relative to an appropriate control (2ΔCt ×100, where ΔCt represents the difference in threshold cycle between the control and target genes).

For microarray analyses, gene expression was ascertained with a MouseRef-8v2.0 BeadChip (Illumina). Probes below background level (detection P-value < 0.01) were excluded, and differential expression was identified with a student’s t-test with Bonferroni correction. Hierarchical clustering and visualization were performed with Cluster 3.0 and TreeView (Eisen software, UC Berkeley). The datasets presented in this study can be found in the online repository Gene Expression Omnibus (GEO), https://www.ncbi.nlm.nih.gov/geo/, accession number GSE161923.

### Flow Cytometry and Sorting

Single-cell suspensions from tumors or cell culture were incubated with FcR blocking IgG for 15 min at 4°C in the dark, to block non-specific staining, then cells were incubated with the appropriate antibodies for 30 min at 4°C in the dark. Then, cells were washed with FACS buffer [PBS (Mediatech; Cat# 21-040-CV) containing 2% heat-inactivated Fetal Bovine Serum (Omega Scientific; Cat# FB-21), 1mM EDTA (Invitrogen; Cat# 15575-038) and 1% Pen-Strep (Gibco; Cat# 15140-122)]. Finally, cells were stained with DAPI (Novus Biologicals; Cat# NBP231156) for 10 min at 4°C in the dark, to allow exclusion of non-viable cells, and washed with FACS buffer.

To quantify the infiltration of NK-92MI cells (GFP-expressing) into SCC-4 or UM-SCC-103 cell-derived tumors, we determined the percentage of GFP^+^CD56^+^ cells in the tumor single-cell suspensions by flow cytometry. To quantify the infiltration of mouse NK cells (labeled with Vybrant Dil-labeling solution) into MOC2 cell-derived tumors, we determined the percentage of NK1.1^+^Vybrant Dil^+^ cells in the tumor single-cell suspensions by flow cytometry. The flow cytometry gating strategy is shown in **Supplementary Figure 3**.

### Cell Migration Assay and Time-Lapse Imaging

NK-92MI cells (10,000 cells in RPMI1640 media) were mixed with 10% Matrigel (BD Matrigel™ Basement Membrane Matrix, Becton Dickinson) and seeded on glass-bottomed 4-well chambered cell culture slides (MatTek). Cells were placed in a live imaging chamber and incubated at 37°C with humidified air supplemented with 5% CO_2_. Time lapse movies for bright field and fluorescence images were acquired every 3 to 5 min using a Nikon Eclipse Ti-U microscope, equipped with an S Plan Fluor ×20 ELWD objective (Nikon) and a Cool Snap HQ2 CCD camera (Photometrics), controlled by NIS-Elements imaging software (Nikon). Movies were processed and annotated using Image J software (National Institutes of Health).

**Figure 1:**
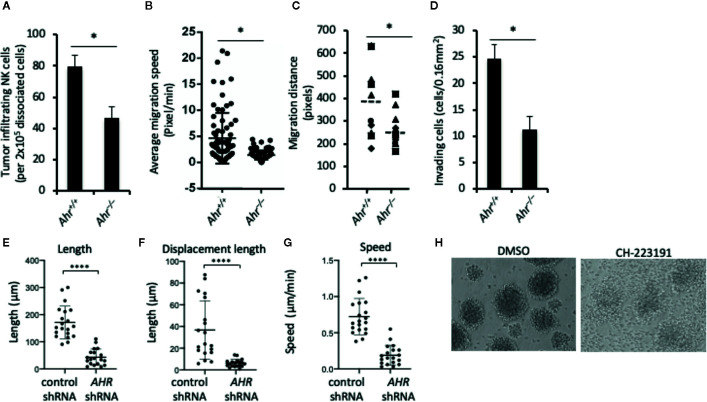
Aryl hydrocarbon receptor (AHR) deficient natural killer (NK) cells have low migratory capacity. **(A)** Tumor infiltration by mouse NK cells. Splenic NK cells from Ahr^+/+^ and Ahr^-/-^ mice were tail vein injected (5x10^6^/mice) into MOC2-bearing NSG mice (n=3). After 24 h, tumors were dissociated, and the amount of NK cells was determined by FACS. Graph is expressed as mean ± SEM. *p < 0.05. **(B, C)** NK cell migration is decreased in AHR-deficient NK cells. Purified NK cells from Ahr^+/+^ and Ahr^-/-^ mice were cultured with IL-2 (1,000 U/mL) for 7 days, then analyzed by time-lapse microscopy. Images were acquired every 5 min for 4 h. **(B)** Average migration speed is presented, 50 cells per group. *p < 0.05. **(C)** Migration distance of Ahr^+/+^ and Ahr^-/-^ NK cells was analyzed with time-lapse microscopy (n=10). **(D)** The invasion ability of Ahr^+/+^ and Ahr^-/-^ NK cells was measured at 24 h after seeding them on the Matrigel chamber (n=10). Results are expressed as the number of invading cells per 0.16 mm^2^. All experiments were repeated at least three times, and the result is expressed as mean ± SEM. *p < 0.05. **(E–G)** NK-92MI cells expressing control shRNA or AHR shRNA were seeded on 5 μg/mL fibronectin. Cells were then imaged in brightfield by live confocal microscopy 2 tp/min and manually tracked. Graphs show the results from one experiment, of three independent experiments, showing length **(E)**, displacement length **(F),** and velocity of cells **(G)** (n=18–20, ****p < 0.0001 by Mann-Whitney test). **(H)** NK-92MI cells were treated with AHR antagonist CH223191 (1uM) or control condition (DMSO) for 2 days, then homotypic aggregation was analyzed using in an optical microscope. One representative example of three different assessments is shown.

For imaging on fibronectin, cells were seeded on #1.5 chambered cell culture slides that had been pre-coated with 5 μg/ml fibronectin. Cells were seeded and allowed to settle for 30 min then images were acquired by LASAF software every 30 s for 2 h using a 1.4 NA 100X objective on a Leica SP8 laser scanning confocal microscope. Data were exported to Fiji (24) and cells were manually tracked. Graphs were generated and statistics performed in Prism 6.0 (GraphPad Software). For the migration assays, NK cells were cultured in IL-2, as indicated in the legend of **Figure 1**.

### Plasmids

Mouse *Asb2* shRNA vector (pLKO.1-GFP-mAsb2) is from GE Healthcare Life Sciences and human *ASB2* shRNA vector (pGIPZ-GFP-Puro-hAsb2) is from openbiosystems. Human filamin A shRNA vector (pLKO.1-hFlna-shRNA) is from GE Healthcare Life Sciences and mouse filamin A shRNA vector (pZIP-UltramiR-mFilamin A shRNA) is from Transomic. Human *AHR* shRNA lentiviral vector (psi-LVRH1GP) was purchased from GeneCopoeia. For mouse *Asb2* expression vector, mouse *Asb2* cDNA fragment (EcoRV/XbaI) from pCMV-SPORT6-mAsb2 (Addgene) was subcloned into lentiviral vector (pLenti-GII-CMV-GFP-2A-Puro; Applied Biological Materials Inc. Canada). Human *ASB2* lentiviral expression vector (pLX304-Blast-V5) is from Gelifesciences. The knockdown efficiency of shRNA was analyzed with qRT-PCR (**Supplementary Figure 4**).

### Invasion Assay

NK-92MI and *ASB2* shRNA stable transfected NK-92MI cells (5×10^5^) were placed in the top well of Matrigel Invasion chambers with serum-free RPMI medium and 0.5 ml of the complete RPMI medium, containing 10% FBS, added to the lower chambers and subjected to invasion assay for 1, 3, or 4 days. Cells were incubated at 37°C for during the timecourse, before the non-invading cells were removed from the upper surface of the membrane. After fixation in 95% ethanol for 5 min, the cells still on the opposite surface of the filter membrane were stained with 1% crystal violet for 10 min. The migratory cells were counted in five microscope fields and averaged.

### Lentiviral Production and Transfection

For the production of the lentiviral particles, the cell line HEK-293 was transfected, using Lipofeactmin2000 (Invitrogen), with the packaging plasmid pCMVR8.74 (addgene), the envelope plasmid pCMV-VSVG and the lentiviral construct containing the shRNA or the transgene. Cell culture medium was changed 16 h after the transfection and virus supernatants were collected 24 and 48 h after the media change. Immediately after supernatant collection, the viral particles were concentrated by ultracentrifugation. The lentiviral pellets were then resuspended in ice-cold PBS and the virus was titrated by FACS using HEK-293 cells.

For the lentiviral transduction of the cell lines, cells were harvested, washed, resuspended in fresh medium and plated at the appropriate concentration (1x10^6^ cells per 10 cm plates). Then, the lentiviral particles were added to the cell cultures at a multiplicity of infection (MOI) of 1 transducing Unit per cell. Polybrene (8 µg/ml) was also added to enhance the lentiviral transduction efficiency. 48 h after viral infection, medium was changed. For cells transduced with the pLKO.1 puro vectors, the cell cultures were treated with the selection agent puromycin for 3 days after media change, all other transfected cells were purified with FACS and amplified for further experiments.

### Western Blot Analysis

NK-92MI cells and primary cultured mouse NK cells were lysed in M-PER mammalian protein extraction reagent (Thermo Scientific; Cat# 78501). Proteins from total cell lysates were resolved on a 4% to 12% Tris-Glycine gel (Life Technologies; Cat# XP04120BOX) and transferred to a polyvinylidene fluoride (PVDF) membrane. Membranes were blocked in TBS containing 0.05% Tween 20 and 5% skim milk powder and incubated overnight with specific rabbit primary antibodies. Secondary donkey-anti-rabbit antibodies conjugated to HRP (Biolegend) were used for chemiluminescence detection with Pierce ECL Western blotting substrate (Thermo Scientific; Cat# 32209). Antibodies to Filamin A was obtained from Antibodies-online. Polyclonal antibody for human Asb2 was purchased from abeomics (Ca# 11-8110).

### Ubiquitination Assay

FICZ treated NK-92MI cells extracts were prepared and immunoprecipitated using the Dynabeads protein G immunoprecipitation kit (Invitrogen; Cat# 10007D). Filamin A antibodies were bound to Dynabeads protein G, and Dynabeads-antibody complex was used to precipitate target proteins from the cell extracts. Unbound proteins were washed away, and complexes were eluted. After mixing with the loading buffer, samples were separated by electrophoresis on SDS-polyacrylamide gels and transferred to PVDF membranes, and Western blotting was performed with ubiquitin antibody (P4D1, Biolegend). MG132 (Selleckchem; Cat# S2619) was used for proteasomal inhibition assay.

### Statistics

For statistical comparison between groups, paired two-tailed Student t-test was used. Analyses were performed using the statistics tools of Microsoft Excel. Mean values are shown unless otherwise indicated. Errors and error bars represent SEM unless otherwise stated. Differences with *p* values <0.05 are considered significant.

## Results

### AHR Regulates the Migration of NK Cells

Consistent with our original observation that AHR deficiency results in poor infiltration of lymphocytes into the tumor microenvironment (18), we found that adoptively transferred murine *Ahr* ^–/–^ NK cells infiltrated tumors to a lower extent than wild-type (WT) NK cells **(** **Figure 1A** **)**. Similarly, time-lapse microscopy of NK cells in an *in vitro* 3D extracellular matrix (ECM)-like culture system showed that *Ahr* ^–/–^ NK cells had lower migration speed, lower migratory distance and lower invasion capacity compared to WT NK cells **(** **Figures 1B–D** **)**. Further, *AHR* shRNA expressing human NK cells (NK-92MI cells) had reduced *in vitro* migratory capacity on fibronectin-coated plates as measured in terms of length, displacement and speed, compared to control cells **(** **Figures 1E–G** **)**. In line with decreased migration and displacement, we found that AHR blockade by its antagonist CH-223191 inhibited NK cell multicellular aggregates **(** **Figure 1H** **)**, which form in response to NK cell activation (25, 26) and involve receptor-ligand interactions and actin dynamics (27). Thus, it appears that AHR activity has an overall effect on NK cell migration capacity.

### *Asb2* Is Regulated by AHR

**Figure 2:**
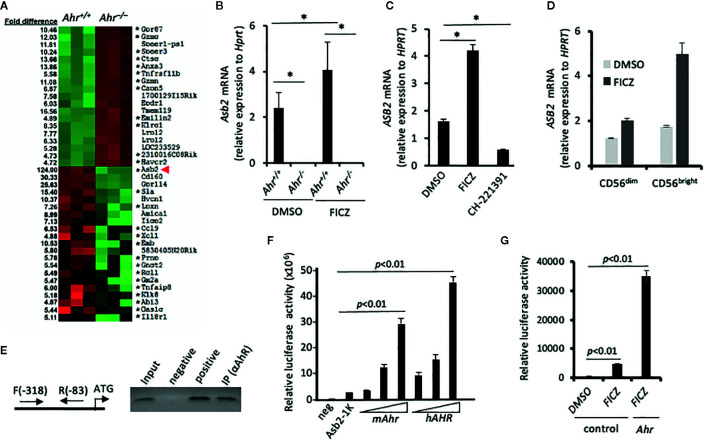
Aryl hydrocarbon receptor (AHR) regulates expression of ubiquitin ligase subunit ASB2. **(A)** Splenic natural killer (NK) cells from Ahr^+/+^ and Ahr^-/-^ mice were cultured in the presence of IL-2 (1,000 U/mL) for 8 days. Then, their gene expression was assessed by microarray. Heatmap shows microarray signal intensity of the top 40 differentially expressed genes (n=3 mice per group). Asterix (*) denotes genes with at least one AHR binding domain in their regulatory region. **(B)** Asb2 mRNA expression was assessed on mouse splenic NK cells cultured in the presence of IL-2 (1,000 U/mL) and FICZ (200 nM) or vehicle control (DMSO) for 7 days (n=3 mice per group). **(C)** Human NK-92MI cells were cultured with AHR agonist FICZ (200 nM), antagonist CH-223191 (1 uM), or vehicle control (DMSO) for 24 h, then Asb2 mRNA expression was determined. **(B, C)** Graphs are expressed as mean ± SEM. *p < 0.05. **(D)** Sorted human CD56dim and CD56bright NK cells were cultured with AHR agonist FICZ (200 nM) or vehicle control (DMSO) for 24 h, then ASB2 mRNA expression was determined. **(E)** Chromatin Immuno-Precipitation assay analyzing the binding of AHR to the promoter region of Asb2 gene. DNA fragments were immunoprecipitated using anti-AHR or anti-H3 antibodies (positive control), or protein A bead only as a negative control; and the immunoprecipitated DNA fragments were amplified using corresponding gene-specific promoter primers. Input: PCR performed using DNA before immunoprecipitation. ATG represents the location of start codon. Arrows labeled F and R represent the gene-specific primers and their positions on target gene promoter. All ChIP experiments were repeated at least three times. **(F)** AHR-dependent increase of Asb2 promoter activity. pBV-Luc-Asb2-1K (-973/-1 from ATG) vector was transfected into HEK-293 cells with mAhR or hAhR expression vectors, and the luciferase activity was measured after 48 h with a Luminometer. Experiments were performed three times. For statistical comparisons, paired two-tailed Student t-test was used. **(G)** FICZ increases Asb2 promoter activity. pBV-Luc-Asb2-1K vector and mouse AhR expression vector were transfected into HEK-293 cells in combination, and 1 day later, HEK-293 cells were treated with FICZ (200 nM) for 24 h. Cells were harvested and luciferase activity was measured with a Luminometer. pBV-Luc-Asb2-1K vector-transfected cells treated with DMSO or FICZ (200 nM) were used as control. Experiments were performed three times. For statistical comparisons, paired two-tailed Student t-test was used.

To elucidate the mechanism of the AHR-mediated regulation of migration, we performed a microarray analysis to assess the differentially expressed genes between *Ahr* ^+/+^ and *Ahr* ^–/–^ NK cells. Strikingly, among the top 40 differentially expressed genes, we observed that the expression of *Asb2*, which encodes the specificity subunit of a multimeric E3 ubiquitin ligase complex (23, 28, 29), had a 124-fold reduction in *Ahr* ^–/–^ NK cells in contrast to the 5 to 15-fold difference observed in other genes **(** **Figure 2A** **)**. A previous study suggested that the *Asb2* promoter contains AhR-binding sites within its regulatory region (12), so we further investigated whether AhR directly regulates *Asb2* expression. Consistent with the microarray analysis, we found that *Ahr* ^–/–^ NK cells had significantly reduced expression of *Asb2* compared to WT NK cells and that the AhR agonist FICZ significantly increased *Asb2* expression in WT NK cells but not in *Ahr* ^–/–^ NK cells **(** **Figure 2B** **)**. We also observed that inhibition of *AHR* expression in human NK cells by shRNA significantly reduced expression of *ASB2* mRNA and protein, compared to control NK cells, indicating an NK cell-intrinsic effect of AHR on ASB2 expression (**Supplementary Figures 1a-b**). Further, we observed that stimulation of human NK cells by FICZ significantly increased *ASB2* expression, while culture of NK cells with the AHR antagonist CH-223191 significantly reduced *ASB2* expression **(** **Figure 2C** **)**. In a previous study, we determined that AHR regulates the function of human CD56^bright^ NK cells, which have a higher expression of *AHR* compared to CD56^dim^ NK cells (19). In line with this, we observed that FICZ induced a marked increase in the expression of *ASB2* in CD56^bright^ NK cells, while only a modest increase in CD56^dim^ NK cells **(** **Figure 2D** **)**. We have previously shown that activating cytokines, such as IL-2, IL-15, and IL-12, upregulate AhR in NK cells (18), and similarly, these cytokines upregulated Asb2 expression, as well (**Supplementary Figure 1b**).

To investigate if *Asb2* expression is directly regulated by AHR, we performed chromatin immunoprecipitation (ChIP) assays using soluble chromatin fragments isolated from IL-2 activated NK cells. PCR amplification of DNA fragments pulled down by anti-AHR antibodies confirmed that AHR bound to genomic regions near the start site of *Asb2* **(** **Figure 2E** **)**. Next, we performed reporter assays in which luciferase expression, and hence activity, was controlled by the *Asb2* promoter region. Co-transfection with either mouse *Ahr* or human *AHR* increased luciferase activity in a dose-dependent manner **(** **Figure 2F** **)**. Furthermore, stimulation by AHR agonist FICZ, particularly in the context of co-transfection with *Ahr*, resulted in increased luciferase activity **(** **Figure 2G** **)**, supporting a direct role of AHR in the control of *Asb2* expression. Taken together, these results indicate that AHR binds to the *Asb2* promoter region and positively regulates *Asb2* expression.

### ASB2 Is Involved in the Regulation of NK Cell Migration

**Figure 3:**
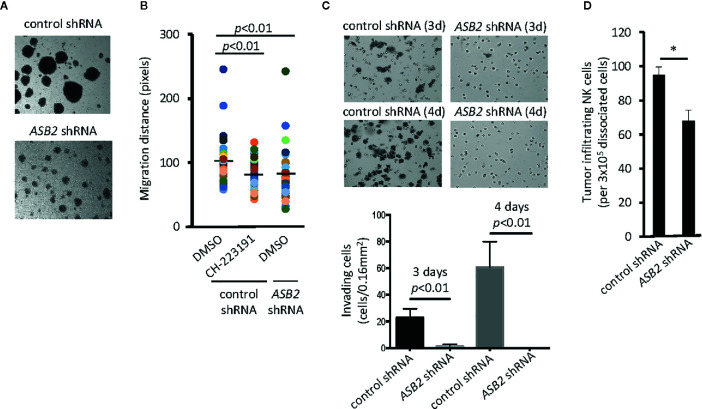
ASB2 is involved in the aryl hydrocarbon receptor (AHR)-mediated regulation of natural killer (NK) cell migration. **(A)** The phenotype of *ASB2* knocked-down or control NK-92MI cells was analyzed using in an optical microscope. One representative example of three different assessments is shown. **(B)** Migration ability of NK cells was analyzed using time-lapse video microscopy. Representative graph shows migration distance of 50 randomly selected NK-92MI cells expressing *ASB2* shRNA or control shRNA, in the presence of CH-223191 (1 μM) or vehicle control (DMSO), on a 10% Matrigel chamber. The quantitation of migration distance was done for 6 h, with 5 min intervals. One representative example of three different assessments is shown. **(C)** Cell invasion was analyzed using *ASB2* knocked-down or control NK-92MI cells. The invasion capacity was measured on day 3 and 4 after seeding. (top) Images show results from a representative experiment; (bottom) graph shows cumulative results from 3 independent experiments expressed as mean ± SD. **(D)** Tumor infiltration by NK-92MI cells. NK-92MI-GFP cells expressing *ASB2* shRNA or control shRNA were tail vein injected (3x10^6^/mice) into UM-SCC-103-bearing NSG mice (n=3). After 24 h, tumors were dissociated, and the amount of infiltrating NK cells was determined by FACS. Graph shows the amount of infiltrating NK cells per 3x10^5^ dissociated cells and is expressed as means ± SEM. *p < 0.05.

Due to the dramatic reduction of *Asb2* expression in *Ahr*-deficient murine NK cells (**Figure 2A**), we investigated whether ASB2 is involved in the regulation of NK cell migration by the aryl hydrocarbon receptor. Using human NK cells, we observed that *ASB2* knockdown resulted in a phenotype similar to *AHR*-deficient NK cells in terms of their ability to form multicellular aggregates in culture **(** **Figures 3A** and **1H** **)**. Using an *in vitro* 3D migration system, we found that both *ASB2*-deficient NK cells and NK cells treated with the AHR inhibitor CH-223191 migrated significantly less compared to control NK cells **(** **Figure 3B** **)**. *ASB2*-deficient NK cells also had a significantly lower capacity to invade Matrigel membranes *in vitro*, compared to control NK cells **(** **Figure 3C** **)**. Finally, we assessed tumor infiltration of adoptively transferred NK cells and found that *ASB2*-deficient NK cells had a decreased ability to infiltrate tumors *in vivo* using a xenograft tumor model **(** **Figure 3D** **)**. Overall, these results demonstrate that ASB2-deficiency phenocopies the AHR-deficiency in NK cells in terms of the ability of NK cells to migrate and infiltrate tumor tissue, thereby providing support for ASB2’s involvement in the AHR-mediated regulation of NK cell migration.

### ASB2 Regulates the Amount of Filamin A

**Figure 4:**
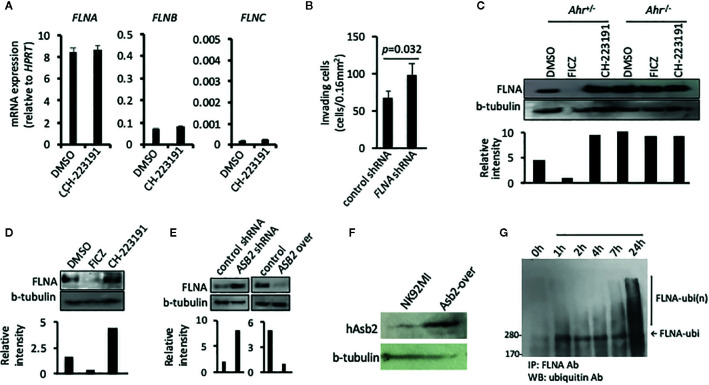
ASB2 regulates filamin A protein level in natural killer (NK) cells. **(A)** NK-92MI cells were cultured in the presence of CH-223191 (1 μM) or vehicle control (DMSO) for 2 days, then the amount of *FLNA*, *FLNB*, and *FLNC* mRNA was determined by qRT-PCR. Graphs show mRNA expression, measured in triplicates, and are shown as mean ± SEM. **(B)** The invasion ability of NK-92MI cells stable transfected with *FLNA* shRNA or control shRNA, was measured at 24 h after seeding on the Matrigel chamber. Results are expressed as the number of invading cells per 0.16 mm^2^. All experiments were repeated at least three times, and the result is expressed as mean ± SEM. *p* value is shown. **(C)** NK cells from *Ahr^+/-^* and *Ahr^-/-^* mice were cultured with IL-2 (1,000 U/ml) for 7 days and then cultured in the presence of FICZ (200 nM), CH-223191 (1 μM), or vehicle control (DMSO) for two additional days. FLNA level was analyzed by western blotting. **(D)** B6 mice were injected intraperitoneally with FICZ (50 μM/mice), CH-223191 (500 μM/mice) or vehicle control (DMSO). After 2 days, the spleens were collected and CD8^-^NKp46^+^ NK cells were FACS sorted and cultured with IL-2 (1,000 U/ml) for 2 days. Then cells were assessed for FLNA level by western blotting. **(E)** The level of FLNA was analyzed on NK-92MI stable transfected with *ASB2* shRNA, *ASB2* over-expression, or control vectors by western blotting. **(F)** ASB2 protein expression was confirmed in NK92MI cells transfected to overexpress *ASB2*. **(G)** Ubiquitination analysis. NK-92MI cells were cultured in the presence of FICZ (200 nM). At different time-points during the culture, NK-92MI were collected, lysed, immunoprecipitated with anti-FLNA antibody and blotted with anti-ubiquitin antibody**. (A–F)** Results from one representative experiment are shown, from at least three different experiments.

One of the major protein targets of the multimeric E3 ubiquitin ligase complex, of which ASB2 provides specificity, is filamin A (23, 28, 29). Filamins are major organizers of the actin cytoskeleton, and their concentration is a crucial determinant of stiffness of the actin filament network, cell spreading, cell adhesion, cell invasion, and migration of immune cells (29, 30). Given that ASB2 is involved in AHR-mediated regulation of NK cell migration, we studied whether this regulation implicated filamins. Analysis of filamin expression indicated that filamin A was the filamin isoform with the highest expression in NK cells and that neither AHR blockade nor *ASB2* downregulation had an impact on filamin A, B and C mRNA levels **(** **Figure 4A** and **Supplementary Figure 2a** **)**. In line with a previous study showing filamin A-degradation dependent migration of dendritic cells (30), we found that knockdown of the expression of *FLNA* increased the invasiveness of NK cells *in vitro* **(** **Figure 4B** **)**. Interestingly, consistent with an AHR-mediated negative regulation of filamin A protein expression, we found that *Ahr* ^–/–^ NK cells had a higher amount of filamin A protein compared to *Ahr* ^+/-^ NK cells, and that filamin A levels were increased in *Ahr* ^+/-^ NK cells treated with the AHR antagonist CH-223191 and decreased in *Ahr* ^+/-^ NK cells treated with the AHR agonist FICZ **(** **Figure 4C** **)**. This modulation of filamin A protein levels in NK cells by AHR ligands was observed *in vitro* and *in vivo* **(** **Figure 4D** and **Supplementary Figure 2b** **)**. In a similar manner, knock-down and over-expression of *ASB2* in NK cells resulted in filamin A levels similar to those resulting from AHR inhibition and activation, respectively **(** **Figure 4E** and **Supplementary Figure 2b** **)**. Overexpression of Asb2 protein was confirmed with Western blotting (**Figure 4F**). Consistent with an involvement of Asb2 in the AHR-mediated regulation of filamin A, we found that FICZ had poor effect on ASB2 knocked-down NK cells **(** **Supplementary Figure 2c** **)**. Also, we found that AHR agonist FICZ increased the amount of filamin A ubiquitination in NK cells **(** **Figure 4G** **)**, and that proteasome inhibitor MG132 partially restored the filamin A levels (**Supplementary Figure 2d**). Overall, these results show that AHR and ASB2 control filamin A protein levels by modulating its ubiquitination and proteasome degradation.

### The AHR-ASB2-FLNA Axis Regulates the Migration of NK Cells

**Figure 5:**
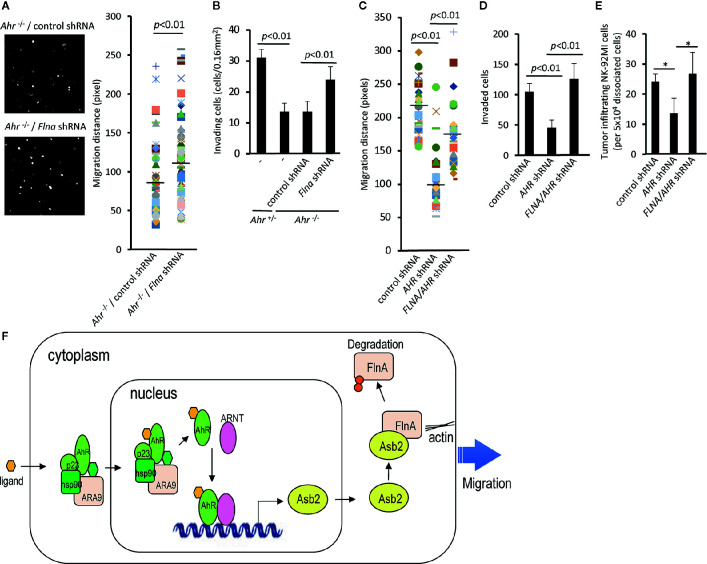
The AHR-ASB2-FLNA axis modulates natural killer (NK)-cell migration. **(A)** Migration of *Ahr^-/-^* NK cells transduced with filamin A shRNA-GFP or control shRNA-GFP vector using time-lapse video microscopy. The quantitation of migration distance was done for 5 h, with 10-min intervals. Graph shows migration distance of 39 randomly selected cells on a 50% matrigel chamber. *p* value is shown. **(B)** NK cells from *Ahr^+/-^* and *Ahr^-/-^* were cultured with IL-2 (1,000 U/mL) for 4 days and transduced with *Flna* shRNA-GFP or control shRNA-GFP vector. Then, FACS sorted GFP^+^ NK cells (2.6x10^5^) were seeded in an invasion chamber. After 24 h, the invasion of NK cells was measured. Results are expressed as the number of invading cells per 0.16 mm^2^. Graph is expressed as mean ± SEM, and *p* values are shown. **(C)** Migration of NK-92MI cells stable transfected with *AHR* shRNA, *FLNA* shRNA, or control vector using time-lapse video microscopy. The quantitation of migration distance was done for 3 h, with 3-min intervals. Graph shows migration distance of 50 randomly selected cells on 10% Matrigel chamber. *p* values are shown. **(D)** NK-92MI stable transfected with *AHR* shRNA, *FLNA* shRNA, or control vector were placed in the top well of a Matrigel invasion chamber (5×10^5^ cells/well). After 2 days, the number of invading cells was determined. Graph is expressed as mean ± SEM, and *p* values are shown. **(E)** Tumor infiltration by NK-92MI cells stable transfected with *AHR* shRNA, *FLNA* shRNA, or control vector. NK-92MI cells were tail vein injected (5x10^6^/mice) into SCC-4-bearing NSG mice (n=3). After 24 h, tumors were dissociated, and the amount of NK-92MI cells was determined by FACS. Graph is expressed as mean ± SEM. *p < 0.05. **(A–E)** One representative example is shown; all experiments were repeated at least three times. **(F)** Model of AhR-mediated regulation of NK-cell migration *via* Asb2-dependent Filamin A degradation.

Our data indicate that AHR-mediated regulation of NK cell migration results from the regulation of filamin A protein levels *via* ASB2. In order to further probe the AHR-ASB2-FLNA axis, we assessed whether filamin A expression knockdown restored the migration capacity of NK cells in the setting of AHR-deficiency **(** **Figure 5** **)**. We found that filamin A knockdown expression in mouse *Ahr^–/–^* NK cells restored their ability to migrate and invade *in vitro* **(** **Figures 5A, B** **)**. Similarly, we observed that knockdown of filamin A expression restored the ability of human AHR knocked-down NK cells to migrate and invade *in vitro* **(** **Figures 5C, D** **)**, as well as their ability to infiltrate tumors *in vivo* **(** **Figure 5E** **)**. Taken together, our results show that AHR modulates the migration of NK cells by regulating the amount of filamin A *via* an AHR-ASB2-FLNA axis **(** **Figure 5F** **)**.

## Discussion

Here, we show that the migration of NK cells is modulated by a previously unrecognized AHR-ASB2-FLNA axis. Our data indicate that AHR directly binds to the *ASB2* promoter and regulates its transcription. In turn, ASB2 regulates the accumulation of filamin A through a ubiquitin-mediated proteasome degradation pathway, which ultimately modulates NK cell migration. While it is possible that AHR and/or ASB2 may be modulating NK cell survival in the tumor instead of migration, our previous extensive analysis of conventional NK cells within AhR-deficient mice did not reveal any appreciable differences in the numbers of conventional NKp46^+^NK1.1^+^CD3^−^CD19^−^ NK cells in the spleens of AhR-deficient mice compared with WT littermates (18). Further, we did not observe baseline differences in the expression of CD27, CD11b, CD117, Ly49, NKG2D, TRAIL, Granzyme A, Granzyme B, or the activation marker KLRG1, suggesting that maturation of conventional NK cells was similar between the mice. Given this, our data indicate that the decrease of the number of conventional NK cells seen in the tumor was likely not due to intrinsic survival of developmental issues of the NK cells, but rather a migration issue. Although it is not clear why filamin A accumulation and failure to be degraded by ASB2 would inhibit NK cell migration, similar observations have been seen in other immune cells. For instance, the lack of filamin A degradation in Asb2-deficient dendritic cells severely affected migration of the dendritic cells (30). In that study, it was proposed that filamin levels need to be tightly coordinated for proper actin-based cell motility.

The mechanisms regulating the migration of NK cells to tissues as well as the ontogeny of tissue-resident NK cells is still a topic of debate. Several populations of tissue-resident NK cells have been described (1, 3). Tissue-resident NK cells are associated with a CD56^bright^ NK cell phenotype (31); in a previous study, we found that CD56^bright^ NK cells highly express AHR and showed that AHR activity can modulate their effector function (19). So, it is possible that AHR may play a role in the migration of these cells into particular tissue microenvironments and that AHR activity is a determinant of tissue-residency.

Given the natural anti-tumoral activity of NK cells, which do not require prior stimulation to kill tumor cells, the number of cancer immunotherapy clinical trials using NK cells has exponentially increased over recent years (32). Solid tumors, in particular immune “cold” tumors, represent a challenge for immunotherapy. Poor lymphocyte-infiltration into those tumors may be multifactorial, including impaired lymphocyte trafficking due to altered cytokine and chemokine secretion (33, 34). Along these lines, it is possible that exclusion of NK cell infiltration into the tumor microenvironment may be due to tumor-derived AHR ligands that affect NK cell migration. It has been shown that certain tumors produce AHR ligands like kynurinine, which is derived from the metabolism of tryptophan by ﻿indoleamine 2,3-dioxygenase 1 (IDO1) (35). Thus, it would be of interest to determine the impact of these tumor-derived AHR ligands on the capacity of NK cell to infiltrate tumors and whether this represents a potential target for cancer immunotherapy.

## Data Availability Statement

The datasets presented in this study can be found in online repositories. The names of the repository and accession number can be found below: Gene Expression Omnibus (GEO), https://www.ncbi.nlm.nih.gov/geo/, GSE161923.

## Ethics Statement

The animal study was reviewed and approved by Stanford University APLAC.

## Author Contributions

Experiments were designed by JHS, LZ, EM, and JBS and were performed by JHS, LZ, CC, AD, and ML. Results were analyzed by JHS, UM-N, EM, and JBS. Manuscript was written by UM-N, JHS, and JBS. All authors contributed to the article and approved the submitted version.

## Funding

This work was supported by funding from the National Institutes of Health (R01CA158516; R35DE030054) to JBS and R01AI137073 to EMM.

## Conflict of Interest

JBS is the scientific co-founder and member of the scientific advisory board of Indapta Therapeutics; however, the science presented here is not related to the focus of the company. UM-N is the founder of Conference Fund; however, the science presented here is not related to the focus of the company.

The remaining authors declare that the research was conducted in the absence of any commercial or financial relationships that could be construed as a potential conflict of interest.

## Supplementary Material

Supplementary Figure 1 Modulations of *ASB2* expression in NK cells. **(A)** Quantitative RT-PCR gene expression analysis of *ASB2* expression in NK92MI cells, transfected either with or without shRNA targeting *AHR*. **(B)** Reduction of ASB2 protein levels after transfection of NK-92MI cells with shRNA targeting *AHR* was confirmed by Western blot. **(C)** *Asb2* expression increases after NK cell activation with cytokines. NK cells were purified from splenocytes of C57BL/6 mice with a negative selection kit and cultured with media containing IL-2 (1x10E3 U/ml), IL-12 (10 ng/ml), IL-15 (10 ng/ml), or IL-18 (10 ng/ml) for 1 hour and 24 hours. Cells were harvested and *Asb2* transcripts were measured by the real-time quantitative RT-PCR assay. All expression levels are normalized to that of HPRT.

Supplementary Figure 2 ASB2 regulates filamin A protein level in NK cells. **(A)** mRNA expression of *FLNA*, *FLNB*, and *FLNC* was determined on NK-92MI stable transfected with *ASB2* shRNA or control shRNA vector, by qRT-PCR. Graphs show mRNA expression, measured in triplicates, and are shown as mean ± SEM. **(B)** NK-92MI cells were cultured in the presence of FICZ (200 nM), CH-223191 (1 uM), or vehicle control (DMSO) for 3 days, then the FLNA protein level was determined by western blotting. **(C)** NK-92MI stable transfected with *ASB2* shRNA or control shRNA were cultured in the presence of FICZ (200 nM) or vehicle control (DMSO) for 3 days, then the FLNA protein level was analyzed by western blotting. **(D)** NK-92MI cells were cultured in the presence of FICZ (200 nM), or vehicle control (DMSO), with or without proteasome inhibitor MG132 (0.1 uM) for 2 days, then the FLNA protein level was analyzed by western blotting. **(A–D)** One representative example is shown; all experiments were repeated at least three times.

Supplementary Figure 3 Flow cytometry strategy of tumor-infiltrating NK cells. **(A)** MOC2 tumors were dissociated, and tail-vein-injected mouse splenic NK cells (stained with Vybrant Dil) were analyzed with anti-NK1.1 antibody. **(B)** UM-SCC-103 tumors were dissociated, and tail-vein-injected GFP-labeled NK-92MI cells were analyzed with anti-CD56 antibody. **(C)** SCC-4 tumors were dissociated, and tail-vein-injected GFP-labeled NK-92MI cells were analyzed with anti-CD56 antibody.

Supplementary Figure 4 Assessment of shRNA knockdown of gene expression. Quantitative gene expression of knocked down genes (human AhR, Asb2 and FLNA) was analyzed by qRT-PCR, using Taqman Gene Expression Assays.
