# Supplementary figures and images for: AHR Regulates NK Cell Migration via ASB2–Mediated Ubiquitination of Filamin A
Source: Front Immunol. 2021 Feb 24;12:624284. doi: 10.3389/fimmu.2021.624284 (PMC7943850; doi:10.3389/fimmu.2021.624284)

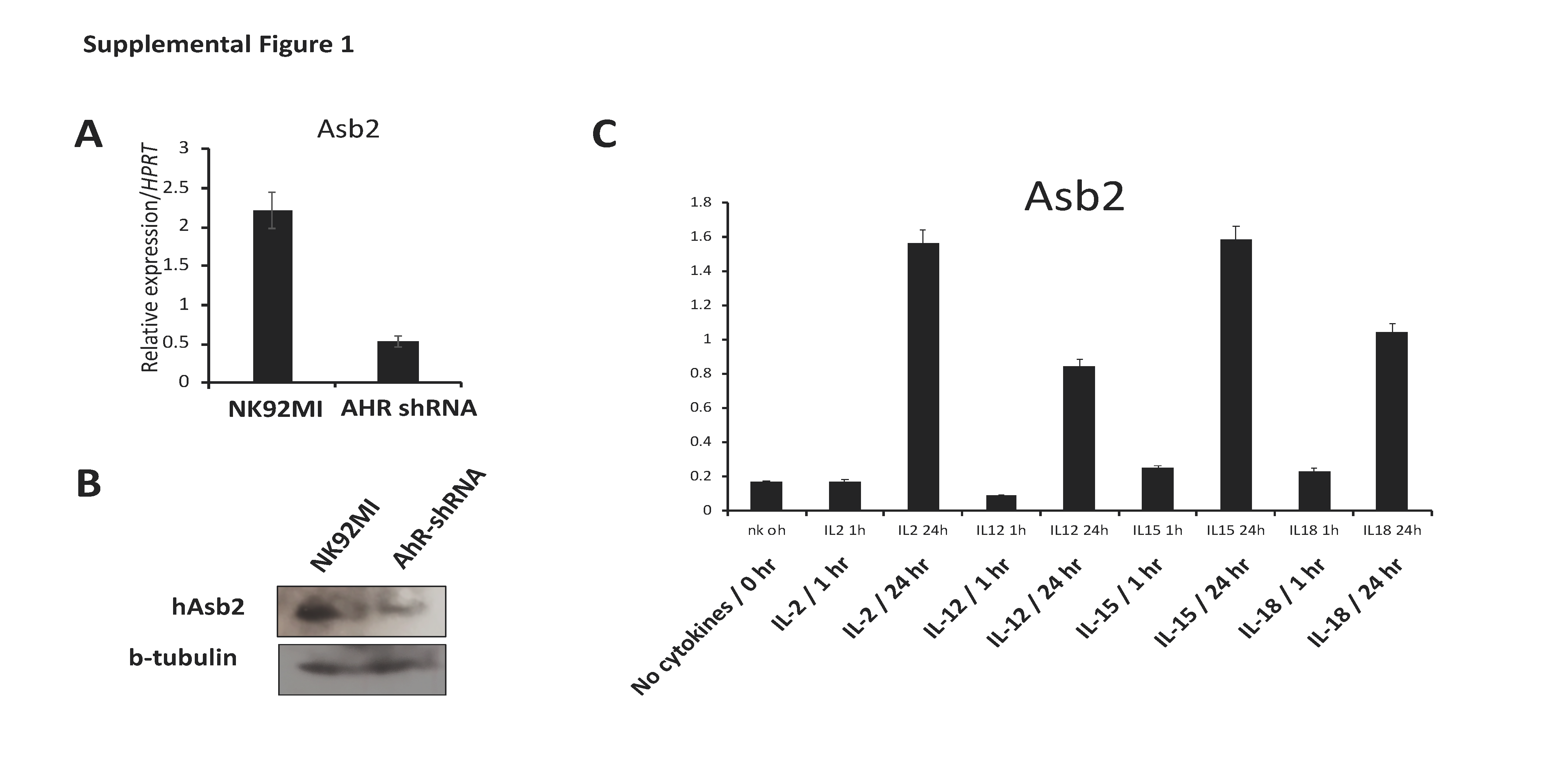

Supplement: Supplementary Figure 1 — Modulations of ASB2 expression in NK cells. (A) Quantitative RT-PCR gene expression analysis of ASB2 expression in NK92MI cells, transfected either with or without shRNA targeting AHR. (B) Reduction of ASB2 protein levels after transfection of NK-92MI cells with shRNA targeting AHR was confirmed by Western blot. (C) Asb2 expression increases after NK cell activation with cytokines. NK cells were purified from splenocytes of C57BL/6 mice with a negative selection kit and cultured with media containing IL-2 (1x10E3 U/ml), IL-12 (10 ng/ml), IL-15 (10 ng/ml), or IL-18 (10 ng/ml) for 1 hour and 24 hours. Cells were harvested and Asb2 transcripts were measured by the real-time quantitative RT-PCR assay. All expression levels are normalized to that of HPRT. [file Image_1.tiff]

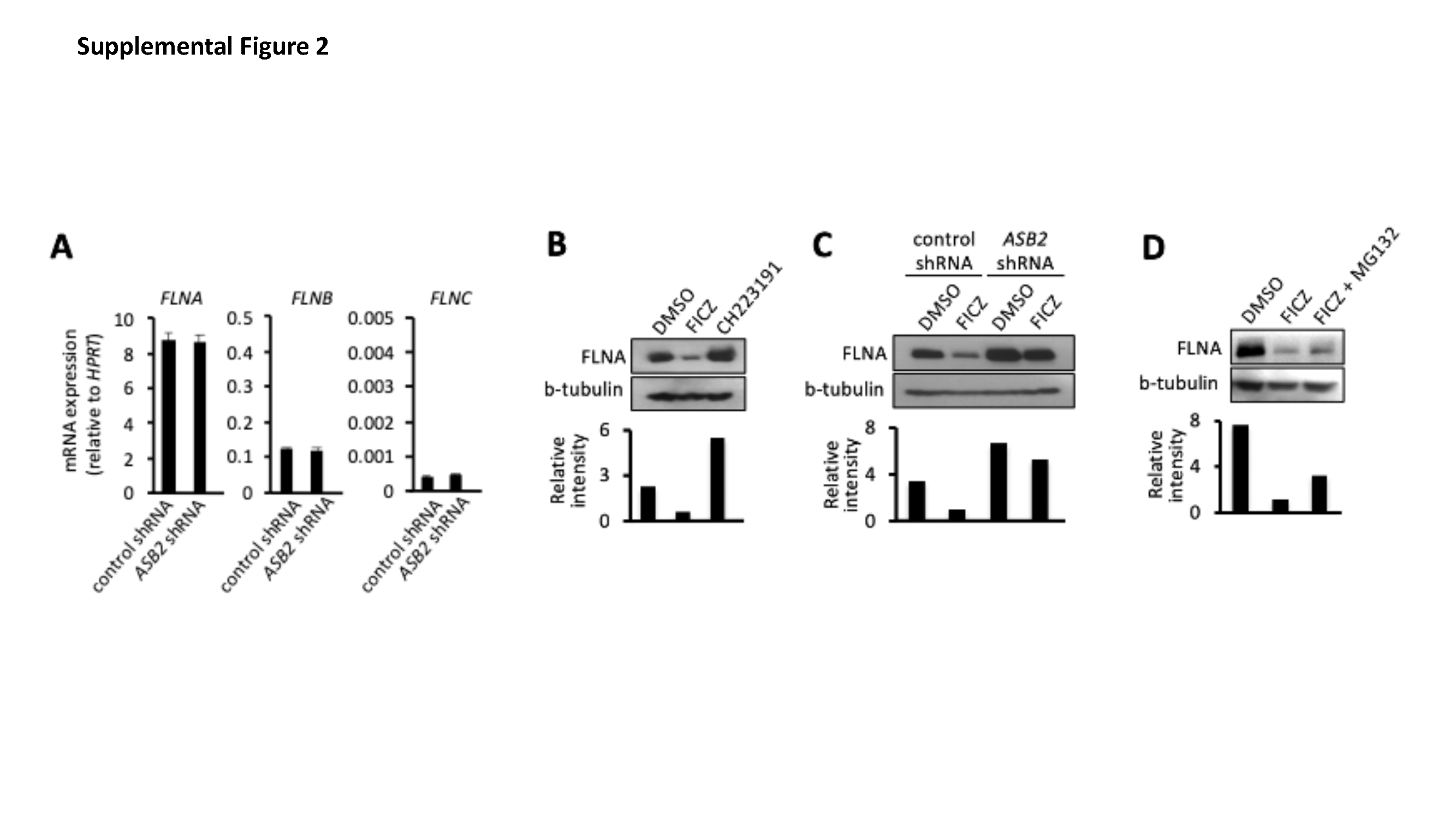

Supplement: Supplementary Figure 2 — ASB2 regulates filamin A protein level in NK cells. (A) mRNA expression of FLNA, FLNB, and FLNC was determined on NK-92MI stable transfected with ASB2 shRNA or control shRNA vector, by qRT-PCR. Graphs show mRNA expression, measured in triplicates, and are shown as mean ± SEM. (B) NK-92MI cells were cultured in the presence of FICZ (200 nM), CH-223191 (1 uM), or vehicle control (DMSO) for 3 days, then the FLNA protein level was determined by western blotting. (C) NK-92MI stable transfected with ASB2 shRNA or control shRNA were cultured in the presence of FICZ (200 nM) or vehicle control (DMSO) for 3 days, then the FLNA protein level was analyzed by western blotting. (D) NK-92MI cells were cultured in the presence of FICZ (200 nM), or vehicle control (DMSO), with or without proteasome inhibitor MG132 (0.1 uM) for 2 days, then the FLNA protein level was analyzed by western blotting. (A–D) One representative example is shown; all experiments were repeated at least three times. [file Image_2.tiff]

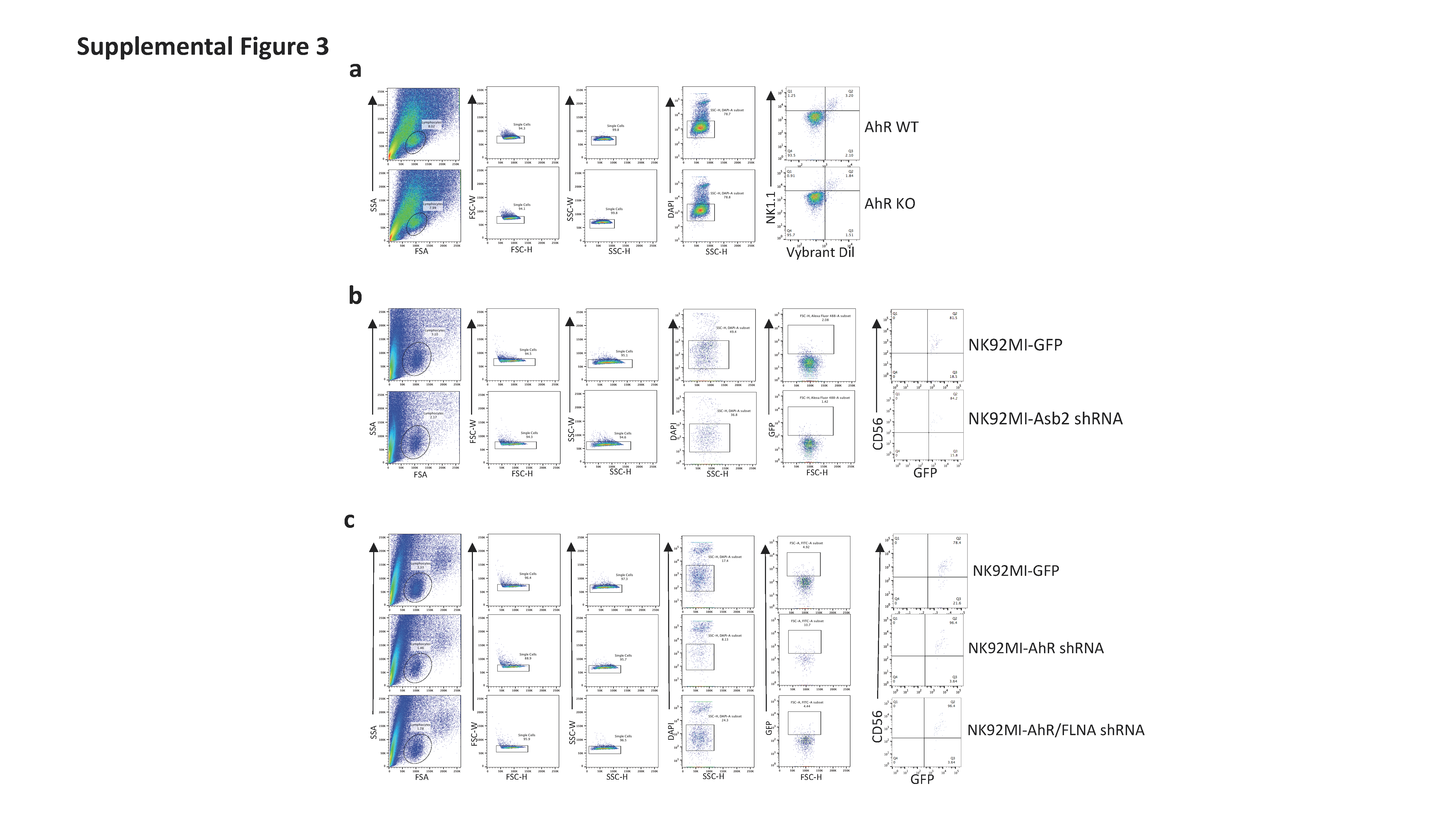

Supplement: Supplementary Figure 3 — Flow cytometry strategy of tumor-infiltrating NK cells. (A) MOC2 tumors were dissociated, and tail-vein-injected mouse splenic NK cells (stained with Vybrant Dil) were analyzed with anti-NK1.1 antibody. (B) UM-SCC-103 tumors were dissociated, and tail-vein-injected GFP-labeled NK-92MI cells were analyzed with anti-CD56 antibody. (C) SCC-4 tumors were dissociated, and tail-vein-injected GFP-labeled NK-92MI cells were analyzed with anti-CD56 antibody. [file Image_3.tiff]

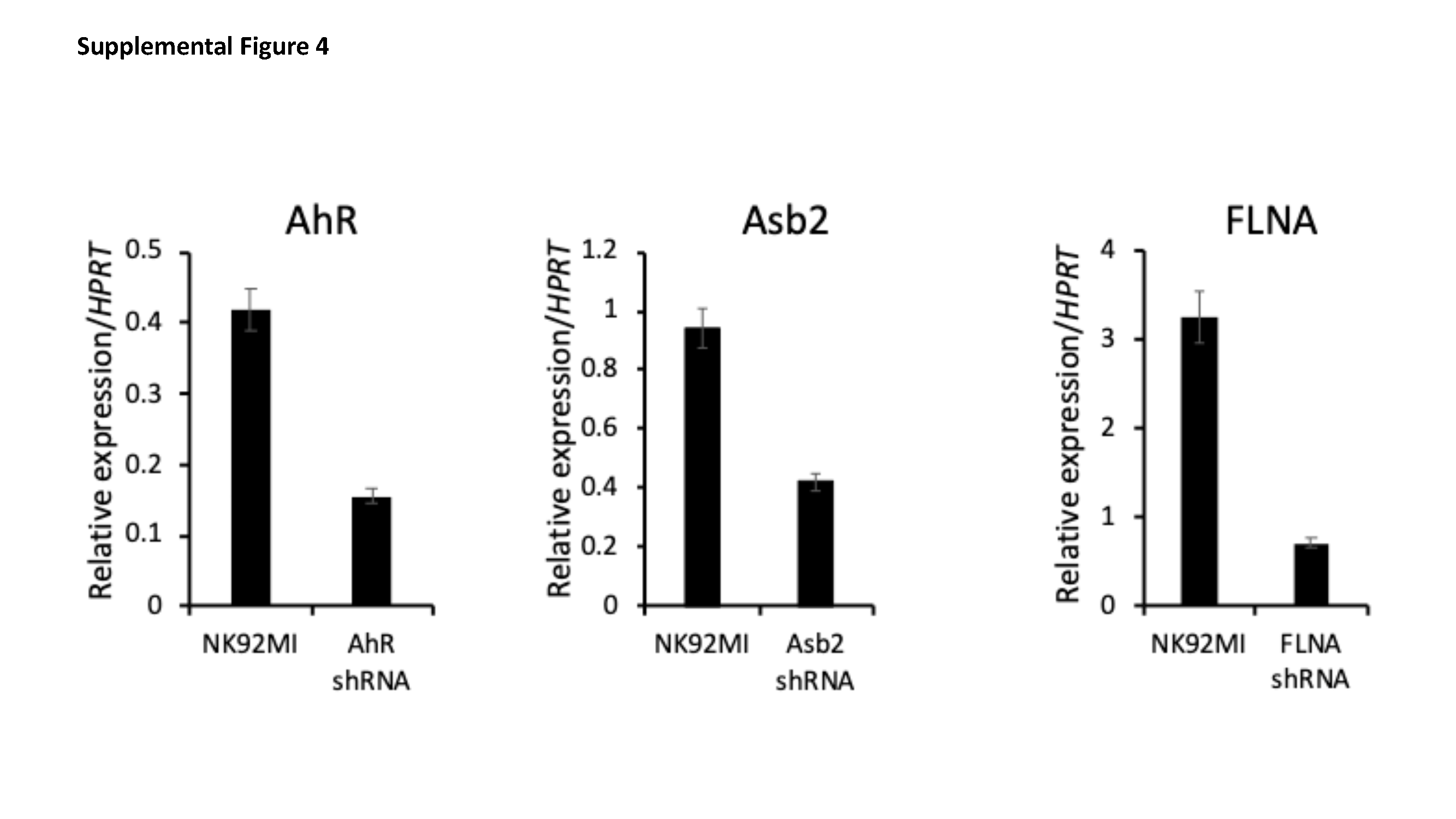

Supplement: Supplementary Figure 4 — Assessment of shRNA knockdown of gene expression. Quantitative gene expression of knocked down genes (human AhR, Asb2 and FLNA) was analyzed by qRT-PCR, using Taqman Gene Expression Assays. [file Image_4.tiff]
